# Dynamic Changes in PD-L1, VEGF, and TILs Following Neoadjuvant Therapy in Residual Invasive Breast Cancer

**DOI:** 10.3390/cimb48090899

**Published:** 2026-09-03

**Authors:** Marina Bakula, Jasmina Rajc, Ana Kvolik Pavic, Slavica Kvolik

**Affiliations:** 1 Clinical Institute of Pathology and Forensic Medicine, University Hospital Center Osijek, Josipa Huttlera 4, 31000 Osijek, Croatia; marina.bakula@kbco.hr (M.B.); jasmina.rajc@kbco.hr (J.R.); 2 Faculty of Medicine, Josip Juraj Strossmayer University of Osijek, Josipa Huttlera 4, 31000 Osijek, Croatia; kvolik-pavic.ana@kbco.hr; 3 Department of Maxillofacial and Oral Surgery, University Hospital Center Osijek, Josipa Huttlera 4, 31000 Osijek, Croatia; 4 Department of Anesthesiology, Intensive Care Medicine and Pain Management, University Hospital Center Osijek, Josipa Huttlera 4, 31000 Osijek, Croatia

**Keywords:** breast cancer, neoadjuvant therapy, PD-L1, VEGF, tumor-infiltrating lymphocytes, triple-negative breast cancer, residual cancer burden, tumor microenvironment

## Abstract

Background/Objectives: Neoadjuvant therapy (NAT) may remodel the breast-cancer microenvironment. We evaluated paired changes in programmed death-ligand 1 (PD-L1), vascular endothelial growth factor (VEGF), and tumor-infiltrating lymphocytes (TILs) before and after NAT, and associations with the residual cancer burden (RCB) and survival. Methods: This retrospective study included 102 patients with residual invasive breast cancer after NAT: 34 with luminal B-like/HER2-negative, 34 with luminal B-like/HER2-positive, and 34 with triple-negative breast cancer (TNBC). PD-L1 was assessed using the 22C3 combined positive score, VEGF by the cytoplasmic staining intensity, and stromal TILs according to international recommendations. Results: The median tumor size decreased from 2.5 to 1.7 cm (*p* < 0.001), the PD-L1 CPS (combined positive score) from 6 to 5 (*p* = 0.039), and the TILs from 15% to 10% (*p* < 0.001), whereas VEGF shifted toward stronger staining (*p* = 0.03). In TNBC, the PD-L1 CPS decreased from 10 to 5 (*p* = 0.003) and the TILs from 20% to 8% (*p* < 0.001). Biomarkers were not associated with the RCB in the overall cohort. The initial tumor size predicted RCB II/III (OR: 2.60, *p* = 0.038). The overall survival differed by subtype (*p* < 0.001). Conclusions: NAT has induced subtype-dependent immune and angiogenic changes, supporting biomarker reassessment in residual disease.

## 1. Introduction

Breast cancer is a heterogeneous disease in which the intrinsic tumor biology, host immunity, and stromal and vascular factors jointly determine the treatment sensitivity and prognosis [1,2]. Neoadjuvant therapy (NAT), initially used primarily to downstage locally advanced disease, is now an integral component of treatment for biologically aggressive breast-cancer subtypes. Beyond increasing the feasibility of breast-conserving surgery, NAT provides an in vivo test of the treatment response and creates a unique paired-sample setting in which pretreatment core-biopsy tissue can be compared with the residual disease at surgery [1,3,4].

The pathologic response after NAT is clinically informative. A pathologic complete response (pCR) is associated with improved long-term outcomes at the patient level, especially in triple-negative breast cancer (TNBC) and HER2-positive disease, although the strength of this association varies among subtypes [5]. Among patients with residual invasive disease, the residual cancer burden (RCB) index provides a reproducible continuous and categorical measure that incorporates the dimensions and cellularity of the residual breast tumor together with nodal involvement [6,7]. The RCB class has been validated as a long-term prognostic measure across breast-cancer subtypes [7,8]. Nevertheless, an anatomic response metric alone cannot fully describe the biological selection and adaptation occurring under treatment pressure.

Programmed death-ligand 1 (PD-L1) is a central immune-checkpoint protein. Its interaction with programmed cell death protein 1 on activated lymphocytes attenuates antitumor immunity, while its expression may also reflect adaptive immune resistance driven by local inflammatory signaling [9,10]. A PD-L1 assessment is clinically relevant in breast cancer, particularly in TNBC, but the results depend on the tumor setting, antibody clone, staining platform, cell population, and scoring algorithm. The 22C3 combined positive score (CPS) integrates PD-L1-positive tumor cells, lymphocytes, and macrophages relative to the number of viable tumor cells. Because PD-L1 is spatially heterogeneous and biologically dynamic, a pretreatment result may not fully represent the residual tumor microenvironment after NAT [11,12,13,14].

Stromal tumor-infiltrating lymphocytes (TILs) provide a morphologic measure of antitumor immune engagement. Higher pretreatment TIL levels are associated with a greater probability of response to NAT and a favorable prognosis in TNBC and HER2-positive breast cancer, although the prognostic meaning of lymphocytic infiltration differs in hormone-receptor-positive disease [15,16]. Paired studies have shown that both PD-L1 and TILs may change after systemic therapy, but the direction and magnitude of change vary across cohorts [11,12,13,14,17]. This variability may reflect the subtype, treatment regimen, sample type, residual-disease selection, and methodological differences in the PD-L1 assessment.

Vascular endothelial growth factor (VEGF) is a major mediator of angiogenesis, vascular permeability, and tumor adaptation to hypoxia [18]. In addition to supporting tumor vasculature, VEGF can suppress dendritic-cell maturation, impair T-cell trafficking and function, promote immunoregulatory cell populations, and increase immune-checkpoint expression [19,20,21]. Thus, VEGF and PD-L1 may represent interacting, though not necessarily linearly correlated, components of an immunosuppressive tumor microenvironment. Breast-cancer studies have reported associations among VEGF, TILs, and PD-L1 [22], while preclinical work provides a biological rationale for combining antiangiogenic and immune-checkpoint approaches [19,20,21]. However, clinical paired-sample data that examine PD-L1, VEGF, and TILs together before and after NAT remain limited.

We therefore investigated paired changes in the PD-L1 CPS, VEGF staining intensity, and stromal TILs between diagnostic core biopsies and residual breast tumors after NAT. The prespecified aims were to: (i) compare baseline biomarker profiles across luminal B-like/HER2-negative, luminal B-like/HER2-positive, and triple-negative tumors; (ii) quantify treatment-associated biomarker changes overall and within each subtype; (iii) examine relationships with the RCB class and residual tumor cellularity; and (iv) explore the overall survival according to the surrogate subtype. We hypothesized that NAT would induce the subtype-specific remodeling of both immune and angiogenic features and that pretreatment biomarker expression would be associated with the extent of residual disease. The primary purpose of this paired analysis was to characterize the treatment-associated biological remodeling of residual breast cancer rather than to evaluate treatment selection or treatment-specific predictive biomarkers.

## 2. Materials and Methods

### 2.1. Study Design and Participants

The Ethics Committee of University Hospital Center Osijek approved the study (No. R1-4425-2/2026). The requirement for informed consent was waived due to the retrospective design and the use of de-identified archival clinical data and tissue samples.

**Figure 1:**
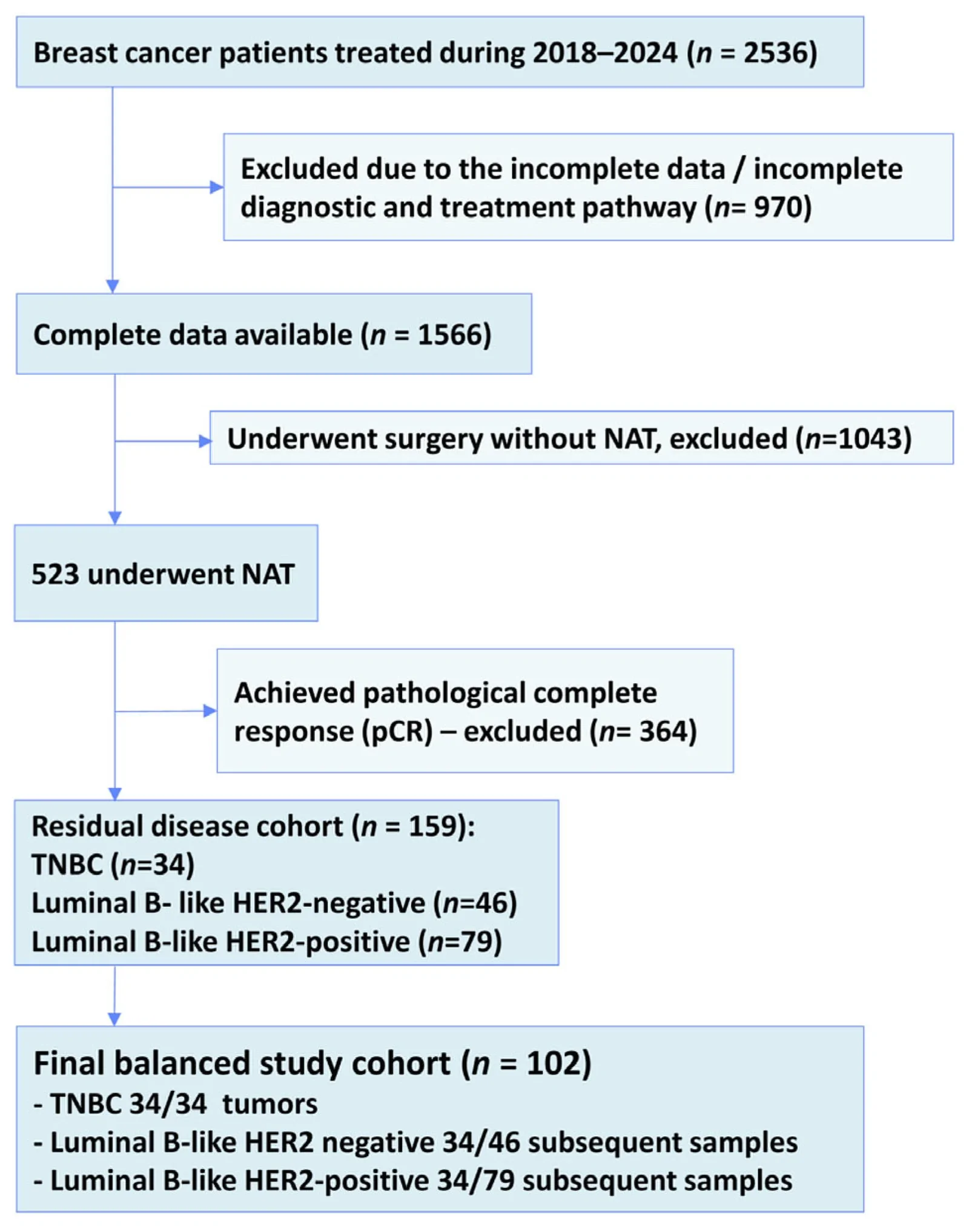
Flowchart of inclusion of the patients with residual invasive breast cancer after NAT for primary breast cancer. NAT: neoadjuvant therapy; TNBC: triple-negative breast cancer.

Patient selection is summarized in Figure 1. Briefly, patients with breast cancer treated during the study period were screened, and those who received neoadjuvant therapy (NAT) were evaluated for eligibility. Cases achieving a pathological complete response after NAT were excluded, as post-treatment residual tumor tissue was not available for the analysis. Only patients with residual invasive disease and paired pre- and post-NAT tissue specimens were included (Figure 1). For the purposes of the study, the first 34 consecutive patients were included in each group to achieve a longer follow-up period for the survival analysis.

To ensure balanced subtype representation, all eligible triple-negative breast-cancer (TNBC) cases were included, and an equal number of consecutive patients from the remaining subtype groups were selected according to the statistician’s recommendation to maximize the follow-up duration. The final study cohort consisted of 102 patients with invasive breast carcinoma who underwent a diagnostic core biopsy, received NAT, and subsequently underwent breast surgery at University Hospital Center Osijek (Figure 1).

The study included three surrogate breast-cancer subtypes, represented in equal proportions: luminal B-like/HER2-negative, luminal B-like/HER2-positive, and TNBC. Paired formalin-fixed paraffin-embedded (FFPE) tissue samples from pretreatment core biopsies and post-NAT surgical specimens were available for all the included patients. Clinical and pathological data were obtained from institutional records. The key inclusion criterion was the availability of complete histopathological reports for both the core needle biopsy obtained before treatment and the breast resection specimen obtained after NAT. All the patients included had residual invasive cancer and were classified as RCB I, II, or III. The exclusion criteria were incomplete data because part of the diagnostic work-up had been performed at other clinical centers, the discontinuation of therapy during NAT, and a pathologic complete response, defined as a resection specimen without residual invasive tumors. Data on the age, tumor size, RCB score, and pathological response were collected from archived histopathological reports held by the Clinical Institute of Pathology and Forensic Medicine. This design permitted a paired biomarker assessment in residual disease, but precluded an evaluation of biomarker associations with a pathologic complete response.

### 2.2. Clinicopathologic Classification and Treatment

The histologic type and grade were determined on routine hematoxylin and eosin (H&E)-stained sections. Hematoxylin and eosin (H&E) staining was performed using hematoxylin and eosin reagents from BioGnost Ltd. (Zagreb, Croatia) on an automated Tissue-Tek® staining system (Sakura Finetek Europe B.V., Alphen aan den Rijn, The Netherlands). Hematoxylin staining was performed for 8 min, followed by eosin staining for 4 min. After staining, the slides were automatically coverslipped using a Tissue-Tek® coverslipping system (Sakura Finetek Europe B.V., Alphen aan den Rijn, The Netherlands) with coverslipping film. H&E-stained sections were subsequently used for the assessment of stromal tumor-infiltrating lymphocytes (TILs). Estrogen receptor (ER) and progesterone receptor (PR) expression were assessed by immunohistochemistry based on nuclear staining in invasive tumor cells. The percentage of positively stained tumor-cell nuclei was recorded from 0% to 100%. ER and PR were considered positive when ≥1% of invasive tumor-cell nuclei showed specific staining and negative when <1% of tumor-cell nuclei were stained. For ER, cases showing staining in 1–10% of tumor-cell nuclei were classified as ER-low positive according to the current ASCO/CAP recommendations [23]. The HER2 status was evaluated by immunohistochemistry and, for equivocal cases, by dual in situ hybridization. HER2 protein expression was assessed by immunohistochemistry based on the intensity and completeness of membranous staining in invasive tumor cells and scored as 0, 1+, 2+, or 3+. An IHC score of 3+ was considered HER2-positive, whereas scores of 0 and 1+ were considered HER2-negative. Cases with an equivocal IHC score of 2+ underwent additional in situ hybridization testing to determine the HER2 gene amplification status; amplified cases were classified as HER2-positive and non-amplified cases as HER2-negative. Surrogate breast-cancer subtypes were defined according to the immunohistochemical phenotype based on protein expression as follows: luminal B-like/HER2-negative tumors were ER- and PR-positive and HER2-negative, with a high Ki-67 proliferation index; luminal B-like/HER2-positive tumors were ER- and PR-positive and HER2-positive; and triple-negative breast cancer (TNBC) was defined as ER-negative, PR-negative, and HER2-negative. The Ki-67 cut-off was defined as <20% for low proliferation and ≥20% for high proliferation.

Neoadjuvant therapy (NAT) was administered in accordance with contemporaneous multidisciplinary recommendations and institutional clinical practice.

For luminal B-like/HER2-negative tumors, neoadjuvant regimens comprised either four cycles of doxorubicin and cyclophosphamide followed by 12 weekly cycles of paclitaxel, four cycles of docetaxel every three weeks, or an aromatase inhibitor for 4–6 months. Patients with luminal B-like/HER2-positive disease received six cycles of docetaxel, carboplatin, trastuzumab, and pertuzumab, or doxorubicin and cyclophosphamide followed by a taxane combined with trastuzumab and pertuzumab. The triple-negative breast-cancer (TNBC) regimens included paclitaxel and carboplatin followed by doxorubicin plus cyclophosphamide or epirubicin plus cyclophosphamide, with or without pembrolizumab. The oncology team always made the final decision on therapy based on the patient’s condition.

### 2.3. Tissue Processing

Core-biopsy specimens were fixed for 24 h in 10% neutral-buffered formalin. Resection specimens were fixed for 24–48 h. After routine processing and paraffin embedding, 4 µm sections were prepared for H&E and immunohistochemical evaluation. Pretreatment and post-NAT slides were reviewed by pathologists with access to the relevant diagnostic material. PD-L1, VEGF, and TIL scoring was performed by a single experienced pathologist who was blinded to the clinical outcomes.

To reduce sampling bias related to intratumoral heterogeneity, resection specimens were serially sampled across the residual tumor bed, and all representative tissue blocks were routinely processed. For the present study, the block containing the largest amount of viable residual invasive carcinoma was selected for biomarker assessment; for the pretreatment core biopsy, the representative block with the highest tumor content was used. PD-L1, VEGF, and TIL assessments were performed in the residual primary breast tumor and not in lymph-node metastases. Nodal disease was included only as a component of the RCB calculation. This approach improved the comparability between paired specimens, although a single selected block cannot fully capture the spatial heterogeneity across a large residual tumor bed.

### 2.4. PD-L1 Immunohistochemistry and Scoring

PD-L1 immunohistochemistry was performed on the Dako Omnis platform (Agilent Technologies, Glostrup, Denmark) using the mouse monoclonal anti-PD-L1 antibody clone 22C3 (IgG1; ready-to-use, Agilent Technologies, Glostrup, Denmark). Following low-pH antigen retrieval (EnVision FLEX Target Retrieval Solution, Low pH; GV805; Agilent Technologies, Glostrup, Denmark), the primary antibody was incubated for 45 min, and staining was visualized according to the manufacturer’s platform protocol. Placental tissue served as a positive control.

The PD-L1 expression was evaluated using CPS:

CPS = (number of PD-L1-staining tumor cells, lymphocytes, and macrophages/total number of  viable tumor cells) × 100.

At least 100 viable tumor cells were required for evaluation. Although continuous CPS values were used for the principal analyses, CPS ≥ 10 was considered positive for descriptive purposes. Necrotic areas, cellular debris, and non-specific staining were excluded.

### 2.5. VEGF Immunohistochemistry and Scoring

VEGF immunohistochemistry was performed on a Ventana BenchMark Ultra platform (Ventana Medical Systems, Inc., Tucson, AZ, USA) using monoclonal mouse VEGF antibody clone C-1 ( sc – 7269; Santa Cruz Biotechnology, Inc., Dallas, TX, USA) at a 1:100 dilution prepared with Dako REAL Antibody Diluent (S2022; Agilent Technologies, Glostrup, Denmark). Following cell conditioning with CC1 buffer, the primary antibody was incubated for 35 min and visualized with the ultraView Universal DAB Detection Kit (Ventana Medical Systems, Inc., Tucson, AZ, USA). Placental tissue was used as the positive control.

The VEGF expression was assessed semiquantitatively as the intensity of cytoplasmic staining in viable tumor cells: 0, absent; 1, weak; 2, moderate; and 3, strong. Positivity required staining in at least 10% of tumor cells. For comparative analyses, evaluable tumors were grouped as weak, moderate, or strong. VEGF staining was evaluable in all 102 pretreatment and 102 post-treatment specimens.

### 2.6. Stromal Tumor-Infiltrating Lymphocytes

Stromal TILs were assessed on H&E-stained sections according to International TILs Working Group recommendations [24]. The percentage of stromal area within the borders of the invasive tumor occupied by mononuclear inflammatory cells, predominantly lymphocytes and plasma cells, was visually estimated and reported as a continuous value from 0% to 100%, in accordance with International TILs Working Group recommendations [24]. Areas of necrosis, crush artifacts, in situ carcinoma, and tissue outside the invasive tumor border were excluded.

### 2.7. Residual Cancer Burden

The RCB was calculated using the MD Anderson method [6], incorporating the dimensions of the residual tumor bed, overall cancer cellularity, proportion of in situ disease, number of positive lymph nodes, and diameter of the largest nodal metastasis. The RCB was categorized as RCB I (>0 to ≤1.36), RCB II (>1.36 to ≤3.28), or RCB III (>3.28). Because the study evaluated samples with residual invasive disease, no RCB 0 cases were included. For univariable logistic regression, RCB I was defined as a favorable response and RCB II/III as an unfavorable response.

### 2.8. Evaluation of Overall Survival

The overall survival (OS) was calculated from the date of initial diagnosis to death from any cause. Patients who were alive at the last follow-up were censored in January 2026. The vital status was obtained from hospital medical records and was available for 100 of 102 patients. Vital-status data were unavailable for two patients because of incomplete follow-up information.

### 2.9. Statistical Analysis

Categorical variables are presented as counts and percentages and were compared using the χ^2^ or Fisher exact test, as appropriate. Paired categorical distributions were compared with the McNemar–Bowker test or test of marginal homogeneity. Distributional assumptions for continuous variables were examined using the Shapiro–Wilk test. Continuous data are presented as the median and interquartile range (IQR). Between-subtype comparisons used the Kruskal–Wallis test with post-hoc Conover comparisons. Paired pretreatment and post-treatment values were compared with the Wilcoxon signed-rank test, accompanied by the Hodges–Lehmann estimate and a 95% confidence interval (CI) where available.

Spearman’s rank coefficient (ρ) was used to examine the correlations of pretreatment clinicopathological and biomarker variables with the RCB class and residual tumor cellularity. Univariable logistic regression estimated the odds ratios (ORs) for RCB II/III. A multivariable model was not fitted because only 11 patients had RCB I and no additional predictors were significant in the univariable analyses. A receiver operating characteristic (ROC) analysis assessed the initial tumor size as a discriminator of RCB II/III, and the optimal threshold was determined by the Youden index. The OS was estimated by a Kaplan–Meier analysis and compared using the log-rank test. The tests were two-sided, and *p* < 0.05 was considered statistically significant. No adjustment was made for multiple comparisons; therefore, the subgroup and exploratory findings should be interpreted cautiously. Statistical analyses were performed using MedCalc® Statistical Software version 23.4.8 (MedCalc Software Ltd., Ostend, Belgium).

## 3. Results

### 3.1. Patient and Pretreatment Tumor Characteristics

The study included 102 patients, comprising 100 women (98%) and two men (2%). Each surrogate subtype included 34 patients. The median age differed among the subtypes (*p* = 0.015); patients with luminal B-like/HER2-positive disease were older than those with TNBC (66 versus 51 years). TNBC was characterized by a higher proportion of grade III tumors (*p* = 0.004) and the highest median Ki-67 index (70%; *p* < 0.001). The initial ultrasonographic tumor size was comparable among subtypes (*p* = 0.960).

The pretreatment PD-L1 CPS differed substantially by subtype (*p* < 0.001). The median CPS was 10 (IQR: 10–20) in TNBC, 1.5 (0–10) in luminal B-like/HER2-negative tumors, and 0 (0–10) in luminal B-like/HER2-positive tumors. The baseline stromal TIL levels did not differ significantly (*p* = 0.923), although the numerical median was highest in TNBC. The VEGF intensity also differed by subtype (*p* = 0.013): strong staining was observed in 47% of TNBCs compared with 24% of luminal B-like/HER2-negative and 18% of luminal B-like/HER2-positive tumors (Table 1).

### 3.2. Paired Changes After Neoadjuvant Therapy

**Figure 2:**
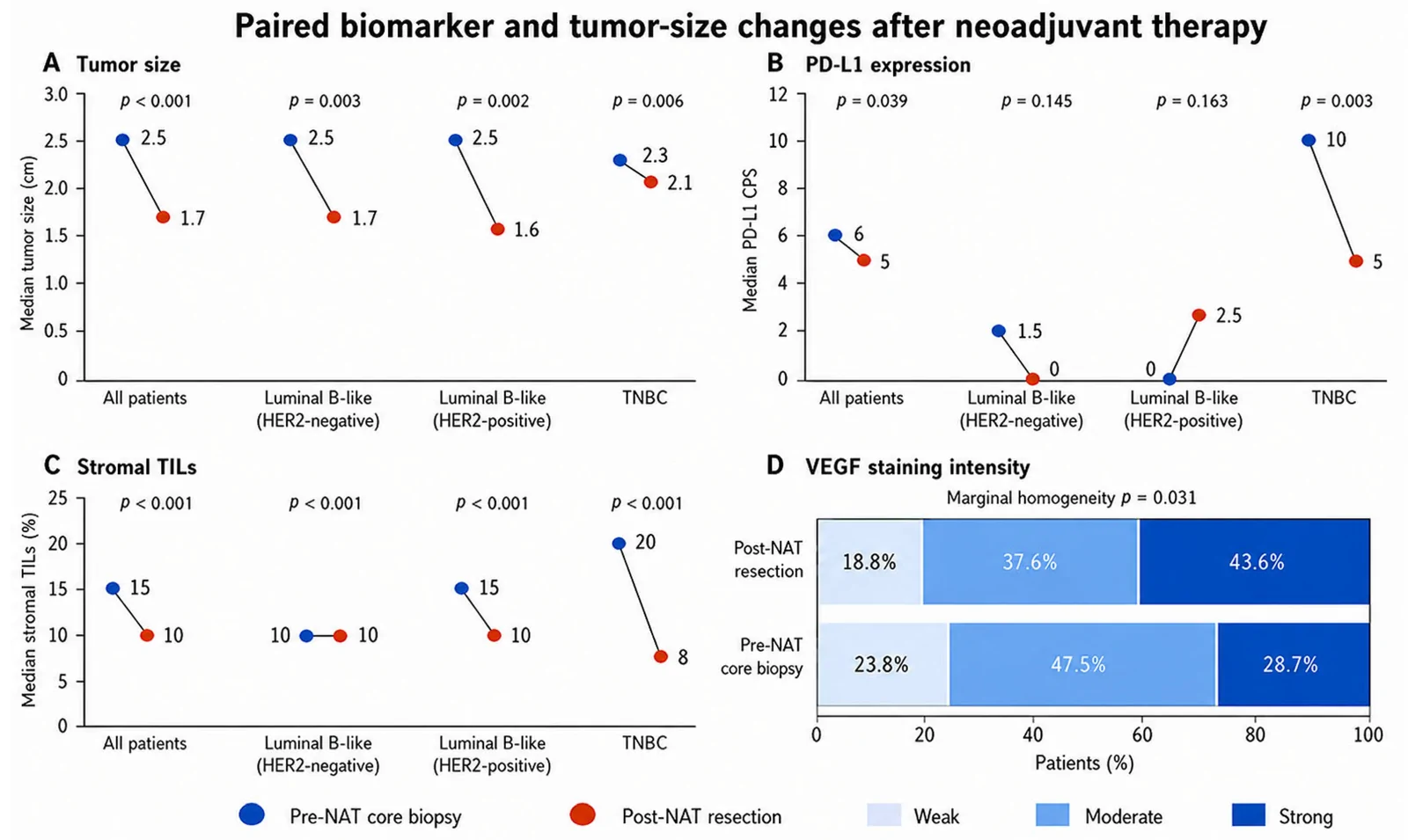
Paired changes in tumor size, PD-L1 CPS, stromal TILs, and VEGF intensity before and after neoadjuvant therapy. Bars show cohort medians for continuous variables and percentage distribution of evaluable cases for VEGF. *p* values are from paired Wilcoxon or paired categorical tests, as appropriate. HER-2, human epidermal growth factor receptor 2; PD-L1, programmed death-ligand 1; TNBC, triple-negative breast cancer; VEGF, vascular endothelial growth factor; NAT, neoadjuvant therapy.

Across the entire cohort, the median tumor size decreased from 2.5 cm (IQR: 2.0–3.1) before NAT to 1.7 cm (1.1–2.3) in the resection specimen (*p* < 0.001). The median PD-L1 CPS decreased modestly from 6 (0–10) to 5 (0–10) (*p* = 0.039), whereas the stromal TILs decreased from 15% (10–40) to 10% (0–10) (*p* < 0.001). The VEGF intensity distributions shifted toward stronger staining after NAT (*p* = 0.03): the proportion of strongly stained tumors increased from 29.4% to 45.1%, while weak and moderate categories declined (Figure 2 and Table 2).

The immune changes were subtype-dependent. The PD-L1 CPS was statistically stable in luminal B-like/HER2-negative and luminal B-like/HER2-positive tumors, whereas TNBC showed a marked decrease from 10 to 5 (*p* = 0.003). The TIL levels decreased significantly in all three subtypes, with the largest estimated median reduction in TNBC. In the TNBC subgroup, the within-patient change in the PD-L1 CPS was not significantly correlated with the change in stromal TILs (Spearman’s ρ = 0.150, *p* = 0.396; *n* = 34). Using the prespecified CPS ≥ 10 threshold, 26/34 (76.5%) TNBC tumors had a CPS ≥ 10 before NAT compared with 12/34 (35.3%) after NAT. Fourteen patients transitioned from a CPS ≥ 10 to a CPS < 10, whereas none transitioned from a CPS < 10 to a CPS ≥ 10 (McNemar test, *p* < 0.001). The tumor size decreased significantly in each subtype; the largest median reduction was observed in luminal B-like/HER2-positive tumors. Additionally, ΔPD-L1 was not significantly correlated with ΔVEGF (ρ = 0.199, *p* = 0.258), whereas ΔTIL was inversely correlated with ΔVEGF (ρ = −0.401, *p* = 0.019).

### 3.3. Residual Disease and RCB

RCB I was present in 11 patients (11%), RCB II in 67 (66%), and RCB III in 24 (24%). The RCB distribution did not differ significantly among the surrogate subtypes (*p* = 0.369) (Table 3). The median continuous RCB index and residual tumor cellularity were also comparable among the subtypes (*p* = 0.869 and *p* = 0.576, respectively). At resection, the PD-L1 CPS and TIL levels no longer differed significantly among the subtypes (*p* = 0.344 and *p* = 0.667).

In the total cohort, the pretreatment tumor size (ρ = 0.331, *p* < 0.001) and clinical T category (ρ = 0.223, *p* = 0.020) were positively correlated with the residual tumor cellularity. In luminal B-like/HER2-negative tumors, the tumor size (ρ = 0.378, *p* = 0.030) and T category (ρ = 0.420, *p* = 0.010) were positively correlated with the residual cellularity, whereas the VEGF intensity was inversely correlated (ρ = −0.444, *p* = 0.010). In TNBC, the pretreatment tumor size was positively correlated with both the RCB class (ρ = 0.350, *p* = 0.042) and the residual cellularity (ρ = 0.499, *p* = 0.001). No significant correlations of the PD-L1 CPS, stromal TILs, or VEGF intensity with the RCB class were observed in the overall cohort or within individual subtypes (Table 4).

An analysis of ΔPD-L1 versus ΔTIL in TNBC was as follows: ΔPD-L1 vs. ΔTIL, ρ = 0.150, *p* = 0.396; ΔPD-L1 vs. ΔVEGF, ρ = 0.199, *p* = 0.258. ΔTIL was negatively correlated with ΔVEGF, ρ = −0.401; *p* = 0.019. In addition, a pre-/post-treatment transition analysis using the prespecified CPS ≥ 10 threshold showed that a pretreatment CPS ≥ 10 was observed in 14/34 (41.2%), and it decreased below 10; no patient with a CPS < 10 transitioned to ≥10, with an exact McNemar *p* < 0.001.

### 3.4. Factors Associated with Greater Residual Cancer Burden

In the univariable logistic regression analysis, the initial tumor size was the only variable significantly associated with RCB II/III (OR per 1 cm increase, 2.62; 95% CI, 1.05–6.54; *p* = 0.039). The age, T category, nodal status, focality, lymphovascular invasion, Ki-67, PD-L1 CPS, stromal TILs, and VEGF intensity were not significantly associated with RCB II/III (Table 5). A multivariable model was not fitted because the favorable-response group comprised only 11 RCB I cases, resulting in an insufficient number of events for a stable adjusted analysis.

The ROC analysis indicated moderate discrimination by the pretreatment tumor size (area under the curve: 0.705, 95% CI: 0.606–0.792; *p* = 0.002). A threshold > 2.6 cm yielded a 45% sensitivity and 100% specificity (Youden index: 0.451). The high specificity should be interpreted cautiously because it was derived from and evaluated in the same small cohort and the sensitivity was limited.

### 3.5. Overall Survival

The vital status was available for 100 patients: 29 deaths were recorded, and 71 patients were alive at the close of follow-up. Deaths occurred in 10 patients with luminal B-like/HER2-negative disease, four with luminal B-like/HER2-positive disease, and 15 with TNBC. The Kaplan–Meier curves differed significantly among subtypes (log-rank *p* < 0.001). The median OS was 31 months (95% CI: 24–55) in TNBC and was not reached in either luminal subgroup. These survival findings should be interpreted cautiously because the analysis was exploratory and unadjusted.

## 4. Discussion

This paired-sample study demonstrates that NAT is accompanied by measurable, subtype-dependent remodeling of immune and angiogenic features in residual breast cancer. Four principal findings emerged. First, the pretreatment PD-L1 CPS and VEGF intensity were highest in TNBC, whereas the baseline stromal TIL levels did not differ significantly among the three equally sized subgroups [25]. Second, across the full cohort, the tumor size, PD-L1 CPS, and TIL levels decreased after NAT, while VEGF staining shifted toward a stronger intensity. Third, the reduction in PD-L1 was driven predominantly by TNBC, in which both the PD-L1 CPS and TILs fell substantially; PD-L1 remained statistically stable in the two luminal groups. Fourth, neither the pretreatment PD-L1, TILs, nor VEGF was significantly associated with the RCB class, while the initial tumor size was associated with a greater likelihood of RCB II/III and with residual cellularity. These findings are interpreted primarily as evidence of treatment-associated biological remodeling of the residual tumor microenvironment rather than as indicators intended to guide a specific treatment decision.

### 4.1. Subtype-Specific Immune Remodeling

The high pretreatment PD-L1 CPS observed in TNBC is consistent with the more immunogenic phenotype of this subtype and with prior reports linking the PD-L1 expression to lymphocytic infiltration and proliferative or aggressive tumor features [9,10,11,12]. Importantly, the paired analysis showed that a single baseline measurement did not remain stable through treatment. The median PD-L1 CPS in TNBC decreased from 10 to 5, while the TILs decreased from 20% to 8%. A plausible interpretation is that the treatment reduced the antigenic tumor mass and depleted or redistributed immune-cell populations contributing to the CPS. Treatment may also select residual clones with a lower checkpoint expression or alter interferon-driven adaptive PD-L1 regulation. Because CPS includes PD-L1-positive immune cells, the concomitant decline in TILs provides a biologically coherent, though not causal, explanation for part of the CPS reduction.

Published paired-sample studies do not show a uniform direction of PD-L1 change. Hoffmann et al. reported heterogeneous changes in PD-L1 and TILs between pretreatment biopsies and post-neoadjuvant surgical specimens, emphasizing assay- and compartment-dependent interpretation [11]. Other cohorts have described decreases, stability, or increases in PD-L1 after therapy [12,13,14,17]. Woo et al., for example, observed increased SP142 immune-cell PD-L1 positivity after neoadjuvant chemotherapy in a subset of TNBC [14]. That result does not necessarily contradict ours: SP142 immune-cell scoring and the 22C3 CPS are not interchangeable, and treatment regimens, sampling, and the composition of residual-disease cohorts differed. Together, these data indicate that the direction of PD-L1 change cannot be generalized across assays and clinical contexts.

The decline in stromal TILs across all subtypes further supports substantial treatment-related immune remodeling. Prior studies have associated higher baseline TILs with a response to NAT, particularly in TNBC and HER2-positive disease [15,16]. However, residual-disease TILs have a more complex interpretation. A low post-treatment TIL level may reflect the effective elimination of immunogenic tumors and the resolution of local inflammation, treatment-related lymphodepletion, or an immune-excluded resistant residual tumor. Conversely, persistent or increased TILs may mark continued immune recognition, but can coexist with checkpoint-mediated dysfunction. Accordingly, a paired change and the post-treatment context may be more informative than an isolated high or low value.

### 4.2. VEGF Dynamics and the Immune–Angiogenic Interface

The shift from weak and moderate toward strong VEGF staining after NAT suggests the enrichment of an angiogenic or hypoxia-adapted phenotype in residual disease. Cytotoxic treatment can disrupt the tumor vasculature and create selective pressure favoring cells capable of surviving hypoxic stress. VEGF may then contribute both to vascular recovery and to an immunosuppressive microenvironment by impairing antigen presentation, limiting effector-cell trafficking, and promoting inhibitory immune programs [18,19,20,21]. This biological framework makes the combined evaluation of VEGF, TILs, and PD-L1 relevant, especially in residual disease.

In the overall cohort, neither the pretreatment PD-L1 CPS nor the VEGF intensity was significantly correlated with the RCB class or residual tumor cellularity. In luminal B-like/HER2-negative tumors, however, a greater VEGF intensity was inversely correlated with the residual cellularity. This isolated subgroup finding should be interpreted cautiously because no adjustment was made for multiple comparisons. Fujii et al. reported relationships among VEGF-A, TILs, and PD-L1 in breast cancer [22], and mechanistic studies support crosstalk between angiogenic and immune-checkpoint pathways [19,20,21]. The present analyses did not directly test the pairwise relationship between the PD-L1 CPS and the VEGF intensity; therefore, no conclusion about their direct correlation or surrogate value can be drawn from these data.

The inverse association between the pretreatment VEGF intensity and the residual cellularity in the luminal B-like/HER2-negative subgroup was unexpected. It may reflect a subtype-specific treatment sensitivity, a chance subgroup finding, or residual confounding by the treatment and tumor features. Because no correction for multiple testing was applied and each subtype contained only 34 patients, this observation is hypothesis-generating and requires external validation.

### 4.3. Biomarkers and Residual Cancer Burden

Neither the pretreatment PD-L1 CPS, the stromal TILs, nor the VEGF intensity was significantly associated with the RCB class. This may initially appear inconsistent with literature linking immune-rich tumors to higher pCR rates [15,16,26]. However, our cohort included only patients with residual invasive disease and contained no RCB 0 cases. Restricting the analysis to the presence of residual disease narrows the response spectrum and may attenuate associations that are most evident when pCR is included. Moreover, RCB I was uncommon, leaving limited statistical power and wide CIs around logistic-regression estimates.

The initial tumor size was the only variable significantly associated with RCB II/III in the univariable analysis and correlated with the residual cellularity overall and within luminal B-like/HER2-negative and TNBC. The OR of 2.62 per 1 cm increase is clinically plausible: larger baseline disease may require a greater absolute cytoreduction to reach minimal residual burden. The ROC threshold > 2.6 cm achieved perfect specificity, but only 45% sensitivity in this cohort. Because the cutoff was selected and tested in the same dataset, it should not be used as a stand-alone clinical decision rule without external validation. The result is better viewed as evidence that the baseline anatomic burden remains relevant even when molecular and immune markers are considered.

Because patients with pCR/RCB 0 have no residual invasive tumors available for a post-NAT biomarker assessment, they could not contribute to the paired component of this study. Therefore, the associations between the baseline tumor size and the RCB reported here apply specifically to patients with residual invasive disease and should not be interpreted as predictors of pCR or of the full spectrum of the neoadjuvant treatment response. RCB categories were used as standardized measures of the residual disease burden rather than as treatment-defining categories; in particular, the distinction between RCB II and RCB III was not intended to imply different post-neoadjuvant treatment strategies. Accordingly, the tumor-size finding is best interpreted as an association between a larger baseline anatomic burden and greater residual disease among patients who did not achieve pCR.

### 4.4. Survival

The observed separation in the OS by subtype, with a median of 31 months in TNBC and the median OS not reached in either luminal group, is consistent with the aggressive clinical course of residual TNBC [5,7,8]. Residual disease after NAT identifies a high-risk population, and the poorer outcome of TNBC underscores the need for effective post-neoadjuvant strategies. Still, the survival analysis was exploratory and unadjusted and did not account for the stage, RCB class, adjuvant therapy, comorbidities, or treatment era. These estimates should therefore be reported descriptively rather than interpreted as an independent prognostic effect.

### 4.5. Clinical Implications

Our results support three practical concepts. First, PD-L1 is biologically dynamic, particularly in TNBC, but its clinical utility depends on the setting. In early-stage TNBC, pembrolizumab is used as part of the neoadjuvant-adjuvant KEYNOTE-522 strategy without selection based on the PD-L1 expression, and the post-neoadjuvant PD-L1 status is not used to determine the continuation of adjuvant pembrolizumab [3,4]. This differs from unresectable locally recurrent or metastatic TNBC, where the PD-L1 expression can play a role in treatment selection depending on the regimen, assay, and validated clinical setting. The ongoing OptimICE-pCR trial evaluates whether adjuvant pembrolizumab can be omitted in patients with early-stage TNBC who achieve pCR after neoadjuvant chemotherapy plus pembrolizumab, emphasizing a pathological response rather than the post-NAT PD-L1 status as the basis of the de-escalation question [27].

In high-risk early HR-positive/HER2-negative breast cancer, PD-L1 testing is not currently established as a routine criterion for treatment selection. Nevertheless, KEYNOTE-756 and CheckMate 7FL provide relevant biomarker correlates: both phase III trials showed higher pCR rates with the addition of immune-checkpoint inhibition to neoadjuvant chemotherapy, with numerically greater treatment effects in tumors with a higher PD-L1 expression [28,29]. These findings support biological enrichment by immune biomarkers, but do not establish post-NAT PD-L1 as a routine treatment-guiding marker in this subtype. Moreover, pCR is less frequent, and its prognostic relationship is more heterogeneous in HR-positive/HER2-negative disease than in TNBC or HER2-positive breast cancer. Thus, the post-NAT PD-L1 changes observed in our cohort should be interpreted as evidence of treatment-associated immune remodeling rather than as an immediately actionable biomarker for adjuvant treatment selection.

Biomarker dynamics, prognostic associations, and treatment-predictive utility are distinct concepts. In conventional oncologic terminology, a predictive biomarker identifies a differential benefit from a specific treatment, whereas a prognostic biomarker is associated with an outcome irrespective of treatment. The present retrospective study did not test treatment-by-biomarker interactions and therefore cannot establish the treatment-specific predictive utility; it evaluated biomarker dynamics and their associations with the residual disease burden. Second, the increase in strong VEGF staining alongside declining TILs suggests that residual disease may become more angiogenic and less immune-infiltrated, supporting the translational investigation of combined vascular-normalizing and immune-directed strategies [19,20,21].

### 4.6. Strengths and Limitations

The principal strength of this study is the paired analysis of three biologically complementary features—PD-L1, VEGF, and stromal TILs—in pretreatment and post-NAT specimens from the same patients. The equal representation of three clinically relevant subtypes enabled subtype-specific comparisons, and the RCB provided a standardized measure of residual disease [6,7,8,30].

Several limitations should be noted. The retrospective, single-center design and small sample size limit the generalizability and statistical power of the findings. Because the study cohort was deliberately balanced to include 34 patients per subtype, it does not reflect the natural distribution of breast-cancer subtypes in the source population. The inclusion criterion requiring residual invasive tumors excluded pCR/RCB 0 cases, introducing selection bias and preventing comprehensive estimates of pCR rates or an assessment of the predictive associations across all response levels. The heterogeneity and incomplete data on NAT regimens hindered the differentiation of treatment-specific effects. Differences between the core biopsy and resection specimens—in tissue volume and potential sampling and heterogeneity issues—may have affected the results. PD-L1 was assessed via the 22C3 CPS, so the findings should not be directly generalized to other clones or scoring methods [11,12,13,31]. The VEGF evaluation was semi-quantitative and did not include measurements such as the microvessel density, circulating VEGF, or immune–vascular spatial relationships. Multiple exploratory comparisons were conducted without correction for multiple testing, and the survival analysis was unadjusted.

The comparison of a limited core-biopsy sample with a substantially larger post-treatment resection specimen is intrinsically vulnerable to spatial heterogeneity. Although representative blocks with the greatest viable tumor content were selected and the resection tumor bed was serially sampled, biomarker values from a single study block may not reflect all heterogeneous regions of residual disease. In addition, the exclusion of pCR/RCB 0 cases was required for the paired post-NAT tumor assessment and limits the generalizability of baseline biomarker-response associations to the full neoadjuvant population.

## 5. Conclusions

NAT was associated with subtype-specific changes in the breast-cancer microenvironment. In residual TNBC, the PD-L1 CPS and stromal TILs decreased significantly, whereas PD-L1 remained statistically stable in luminal B-like/HER2-negative and luminal B-like/HER2-positive tumors. Across the full cohort, the VEGF expression shifted toward a stronger intensity after treatment. The pretreatment PD-L1, TILs, and VEGF were not associated with the RCB class, while a larger initial tumor size was associated with a greater residual disease burden among patients with residual invasive disease. These findings support a paired biomarker assessment as a means of characterizing treatment-induced biological change rather than as evidence of the treatment-specific predictive utility. Prospective, treatment-stratified studies that include the full response spectrum, including pCR, are required before dynamic PD-L1 or VEGF measurements can guide clinical decisions.

## Figures and Tables

**Table 1:** Selected pretreatment characteristics according to surrogate subtype.

Characteristic	Luminal B-like/HER2-Negative (*n* = 34)	Luminal B-like/HER2-Positive (*n* = 34)	TNBC (*n* = 34)	*p* Value
Age, years	57 (51–65)	66 (53–73)	51 (44–63)	0.015 ^‡^
Histologic grade I/II/III, n	6/20/8	7/20/7	0/16/18	0.004
Ki-67, %	37 (25–50)	47.5 (30–78.5)	70 (57.8–90)	<0.001 ^§^
Tumor size, cm	2.5 (1.8–3.2)	2.5 (2.1–3.0)	2.3 (2.0–3.2)	0.960
PD-L1 CPS	1.5 (0–10)	0 (0–10)	10 (10–20)	<0.001 ^ǁ^
Stromal TILs, %	10 (10–40)	15 (10–40)	20 (10–60)	0.923
N0/N1, *n* (*n* = 87)	19/12	11/21	15/9	0.047
VEGF weak/moderate/strong, n	10/16/8	12/16/6	2/16/16	0.013

Data are presented as median (IQR) unless otherwise indicated. Between-group comparisons were performed using Kruskal–Wallis test with post hoc Conover test, chi-square test, or Fisher’s exact test, as appropriate. Significant post hoc differences (*p* < 0.05) were observed between: ^‡^ luminal B-like/HER2-positive and TNBC; ^§^ all three subtypes; and ^ǁ^ luminal B-like/HER2-positive and TNBC. VEGF was assessable in all 102 pretreatment specimens. CPS, combined positive score; TILs, tumor-infiltrating lymphocytes; TNBC, triple-negative breast cancer; VEGF, vascular endothelial growth factor.

**Table 2:** Paired changes in tumor size, PD-L1 CPS, and stromal TILs overall and by subtype.

Group/Variable	Pretreatment	Post-NAT Resection	Hodges–Lehmann Difference (95% CI)	*p* Value
**All patients (** ***n* = 102)**				
Tumor size, cm	2.5 (2.0–3.1)	1.7 (1.1–2.3)	−0.6 (−1.1 to −0.3)	<0.001
PD-L1 CPS	6 (0–10)	5 (0–10)	0 (−5 to 0)	0.039
Stromal TILs, %	15 (10–40)	10 (0–10)	−15 (−20 to −10)	<0.001
**Luminal B-like/HER2-negative (*n* = 34)**				
Tumor size, cm	2.5 (1.8–3.2)	1.7 (1.4–2.3)	−0.6 (−1.1 to −0.3)	0.003
PD-L1 CPS	1.5 (0–10)	0 (0–10)	0 (−5 to 0)	0.145
Stromal TILs, %	10 (10–40)	10 (0–10)	−15 (−23 to −10)	<0.001
**Luminal B-like/HER2-positive (*n* = 34)**				
Tumor size, cm	2.5 (2.1–3.0)	1.6 (1.0–2.2)	−1.0 (−1.3 to −0.7)	0.002
PD-L1 CPS	0 (0–10)	2.5 (0–20)	0 (0 to 5)	0.163
Stromal TILs, %	15 (10–40)	10 (0–20)	−10 (−20 to −5)	<0.001
**TNBC (*n* = 34)**				
Tumor size, cm	2.3 (2.0–3.2)	2.1 (1.2–2.5)	−0.5 (−0.9 to −0.3)	0.006
PD-L1 CPS	10 (10–20)	5 (0–10)	−8 (−15 to −3)	0.003
Stromal TILs, %	20 (10–60)	8 (0–10)	−15 (−25 to −5)	<0.001

Data are median (IQR). Paired comparisons used Wilcoxon signed-rank test. NAT, neoadjuvant therapy; CPS, combined positive score; TILs, tumor-infiltrating lymphocytes; TNBC, triple-negative breast cancer.

**Table 3:** Residual cancer burden class according to molecular subtype.

RCB Class	Luminal B-like/HER2-Negative (*n* = 34)	Luminal B-like/HER2-Positive (*n* = 34)	TNBC (*n* = 34)	Total (*n* = 102)
RCB I	1 (3%)	5 (15%)	5 (15%)	11 (11%)
RCB II	26 (76%)	21 (62%)	20 (59%)	67 (66%)
RCB III	7 (21%)	8 (24%)	9 (26%)	24 (24%)

Fisher exact test, *p* = 0.369. RCB, residual cancer burden; TNBC, triple-negative breast cancer.

**Table 4:** Spearman correlations of pretreatment clinicopathological and biological variables with RCB class and residual tumor cellularity.

Group/Variable	RCB Class, ρ (*p* Value)	Residual Cellularity, ρ (*p* Value)
**All patients**		
Tumor size	0.271 (0.006)	0.331 (<0.001)
T category	0.009 (0.930)	**0.223 (0.020)**
Ki-67	0.026 (0.790)	0.064 (0.520)
PD-L1 CPS	0.034 (0.734)	−0.078 (0.440)
Stromal TILs	0.139 (0.160)	0.111 (0.270)
VEGF intensity	0.014 (0.889)	−0.087 (0.390)
**Luminal B-like/HER2-negative**		
Tumor size	0.153 (0.390)	**0.378 (0.030)**
T category	0.026 (0.880)	**0.420 (0.010)**
Ki-67	−0.182 (0.300)	−0.188 (0.290)
PD-L1 CPS	−0.140 (0.430)	−0.100 (0.570)
Stromal TILs	0.164 (0.360)	0.254 (0.150)
VEGF intensity	−0.138 (0.440)	**−0.444 (0.010)**
**Luminal B-like/HER2-positive**		
Tumor size	0.101 (0.570)	0.056 (0.760)
T category	−0.049 (0.780)	−0.021 (0.900)
Ki-67	0.136 (0.440)	0.076 (0.670)
PD-L1 CPS	0.086 (0.630)	−0.165 (0.350)
Stromal TILs	0.318 (0.070)	0.001 (>0.990)
VEGF intensity	−0.029 (0.870)	0.220 (0.220)
**Triple-negative**		
Tumor size	0.350 (0.042)	0.499 (0.001)
T category	0.099 (0.578)	0.247 (0.159)
Ki-67	0.218 (0.215)	0.132 (0.458)
PD-L1 CPS	0.123 (0.488)	0.052 (0.771)
Stromal TILs	0.019 (0.915)	0.075 (0.674)
VEGF intensity	−0.050 (0.777)	0.083 (0.643)

Values are Spearman’s ρ (*p* value). Bold values indicate *p* < 0.05. RCB, residual cancer burden; TILs, tumor-infiltrating lymphocytes; VEGF, vascular endothelial growth factor.

**Table 5:** Univariable logistic regression analysis for RCB II/III.

Predictor	OR	95% CI	*p* Value
Age ≥ 50 years	1.35	0.37–5.01	0.649
Initial tumor size, per 1 cm	2.62	1.05–6.54	0.039
T2–T3 vs. T1	0.74	0.15–3.69	0.715
N1 vs. N0	1.95	0.34–11.26	0.455
Multifocal vs. unifocal	0.71	0.08–6.47	0.758
Lymphovascular invasion, present	0.58	0.06–5.49	0.636
Ki-67, per 1%	0.98	0.95–1.01	0.108
PD-L1 CPS, per unit	1.01	0.97–1.04	0.798
Stromal TILs, per 1%	1.002	0.98–1.03	0.887
VEGF moderate vs. weak	0.64	0.12–3.42	0.598
VEGF strong vs. weak	0.79	0.12–5.15	0.803

OR, odds ratio; CI, confidence interval; CPS, combined positive score; TILs, tumor-infiltrating lymphocytes; VEGF, vascular endothelial growth factor. Grade estimates were unstable because of sparse cells and are omitted from the display.

## Data Availability

The data supporting the findings of this study are available from the corresponding author upon reasonable request, subject to institutional and ethical restrictions protecting patient confidentiality.

## Abbreviations

The following abbreviations are used in this manuscript:

CPS	combined positive score
ER	estrogen receptor
FFPE	formalin-fixed, paraffin-embedded
HER2	human epidermal growth factor receptor 2
IQR	interquartile range
NAT	neoadjuvant therapy
OR	odds ratio
OS	overall survival
pCR	pathologic complete response
PD-L1	programmed death-ligand 1
PR	progesterone receptor
RCB	residual cancer burden
ROC	receiver operating characteristic
TILs	tumor-infiltrating lymphocytes
TNBC	triple-negative breast cancer
VEGF	vascular endothelial growth factor
